# Natural products chemistry in the third millennium

**DOI:** 10.1186/1752-153X-1-1

**Published:** 2007-02-19

**Authors:** Vassya Bankova

**Affiliations:** 1 Institute of Organic Chemistry with Centre of Phytochemistry, Bulgarian Academy of Sciences, 1113 Sofia, Bulgaria.

## Abstract

The main directions of Natural Products Chemistry in the Third Millennium are summarized.

In the beginning of organic chemistry was natural products chemistry. For a long period, up to the 1960's the structural studies of natural products served as the principle driving force for the discovery of new chemical reactivity. The introduction of spectroscopic techniques, however, removed much of the "intellectual challenge" involved in structure elucidation. Furthermore, natural products chemistry suffered a dramatic decline from the mid 1990's when major pharmaceutical companies disinvested in this area and switched to more "rational" combi-chem approaches. Nevertheless, the improvements in spectroscopic methods have historically stimulated natural products chemistry and the efforts to examine new compounds from unusual organisms rapidly and systematically. Natural products chemistry survived and began to flourish again in recent years also through chemical biology and chemical genetics and the realization that natural product structures often explore structural space unavailable to combi-chem approaches. As a result, challenges for natural product chemists are not diminishing, they are just changing. Natural product chemistry turned to an interdisciplinary science, where the success of a chemist would only be possible in close collaboration with biologists, pharmacologists, and clinicists. Thus many novel biological activities - such as beta-tubulin assembly inhibitors for example, could only have emerged from the natural products arena.

Combined with pharmacological screening, natural products chemistry has always provided highly useful leads for drug discovery. The searches for new biologically active compounds are most often based on hints coming from ethnobotany but there are still a huge number of unstudied plants, not to speak of mushrooms, marine organisms, insects, and microorganisms. There is a wealth of molecular diversity out there, waiting to be discovered and utilized. The central issue of such type of studies, structure elucidation, although often believed to be trivial, is still a process full of adventure, discovery, and even unavoidable pitfalls. Thus structure elucidation has still much to offer, especially when combined with biological tests. *Chemistry Central Journal *is waiting for your results to publish.

Besides the classic studies connected to pharmacological activities, new developments challenge natural products chemists, such as metabolomics, the large-scale phytochemical analysis in the functional genomics era. Metabolomic requires from a natural product chemist brilliant knowledge of modern analytical techniques and chemometry and close collaboration with biochemists and biologists. Chemical ecology, too, could not advance properly without natural product chemistry.

Approximately 60% of the world's population relies almost entirely on plants for medication. However, if phytopharmaceuticals want to be regarded as rational drugs, they need to be standardized and pharmaceutical quality must be approved. For this reason, another important task for natural products chemistry is connected to standardization: to develop proper analytical methods of quality control, to make sure that medicines obtained from natural sources are safe and of reproducible efficacy.

The publication of natural product research results in an open access journal is of great importance with respect both to research activities and to effective use of natural resources, removing both price and permission barriers. It is also important to authors, giving them the opportunity to publish their results where they will be most easily accessed by those who mostly need them. The new open access *Chemistry Central Journal *gives this opportunity to authors and readers in all domains of Natural Product Chemistry.

We look forward to your enthusiastic and frequent participation in the exciting project *Chemistry Central Journal *as readers and authors.

